# Supplementary material for: Identification of small molecule inhibitors of G3BP-driven stress granule formation
Source: J Cell Biol. 2024 Jan 29;223(3):e202308083. doi: 10.1083/jcb.202308083 (PMC10824102; doi:10.1083/jcb.202308083)
Supplement: Table S1 — shows crystallographic statistics for G3BP1 NTF2L domain with G3la. [file JCB_202308083_TableS1.docx]

|  | G3BP1 NTF2L / G3Ia  (PDB: 8V1L) |
| --- | --- |
| **Data collection** | |
| Space group | P2_1_ |
| **Cell dimensions** | |
| a, b, c (Å) | 52.17, 84.69, 102.15 |
| α, β, γ (°) | 90, 91.26, 90 |
| Resolution (Å) | 46.87 – 2.68  (2.75 – 2.68)^a^ |
| Completeness (%) | 96.5 (95.9) |
| **Refinement** | |
| No. reflections | 23085 |
| R_work_/R_free_ | 0.276 / 0.395 |
| **No. atoms** | |
| Protein | 6490 |
| Water | 92 |
| Wilson B-factor (Å^2^) | 34.2 |
| **r.ms.d.** | |
| Bond lengths (Å) | 0.004 |
| Bond angles (º) | 1.179 |
| **Ramachandran statistics (%)** | |
| Favored regions | 85.14 |
| Outliers | 3.65 |

^a^ = highest resolution shell in parenthesis

Table S1: Crystallographic statistics for G3BP1 NTF2L domain with G3Ia
