# Supplementary material for: Identification of small molecule inhibitors of G3BP-driven stress granule formation
Source: J Cell Biol. 2024 Jan 29;223(3):e202308083. doi: 10.1083/jcb.202308083 (PMC10824102; doi:10.1083/jcb.202308083)
Supplement: Supplementary file 12 — SourceData F2 is the source file for Fig. 2. [file JCB_202308083_SourceDataF2.pdf]

Figure 2A

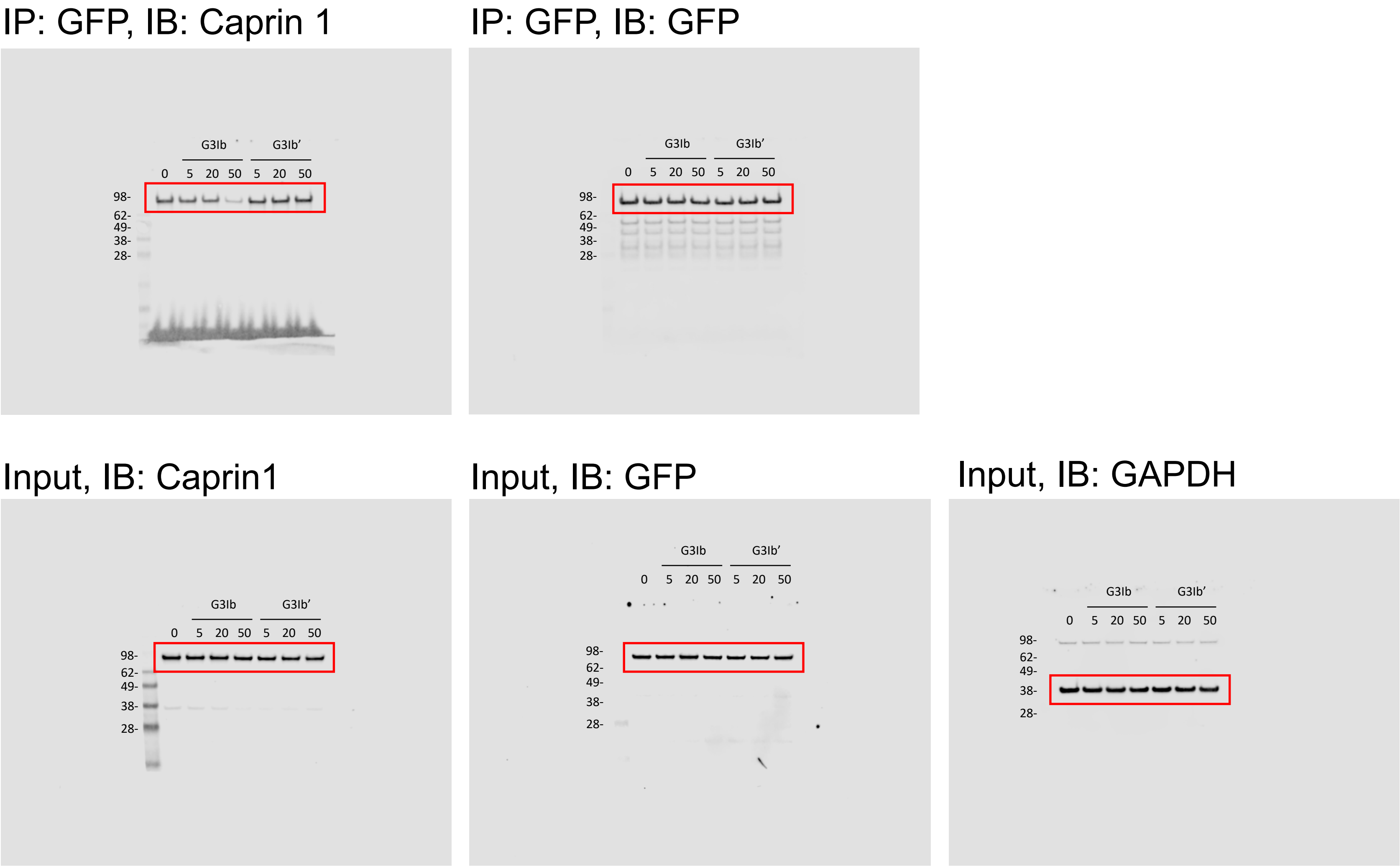
IP: GFP, IB: Caprin 1
G3Ib
G3Ib’
0 5 20 50 5 20 50
98-
62-
49-
38-
28-
IP: GFP, IB: GFP
G3Ib
G3Ib’
0 5 20 50 5 20 50
98-
62-
49-
38-
28-
Input, IB: Caprin1
G3Ib
G3Ib’
0 5 20 50 5 20 50
98-
62-
49-
38-
28-
Input, IB: GFP
G3Ib
G3Ib’
0 5 20 50 5 20 50
98-
62-
49-
38-
28-
Input, IB: GAPDH
G3Ib
G3Ib’
0 5 20 50 5 20 50
98-
62-
49-
38-
28-

Figure 2C

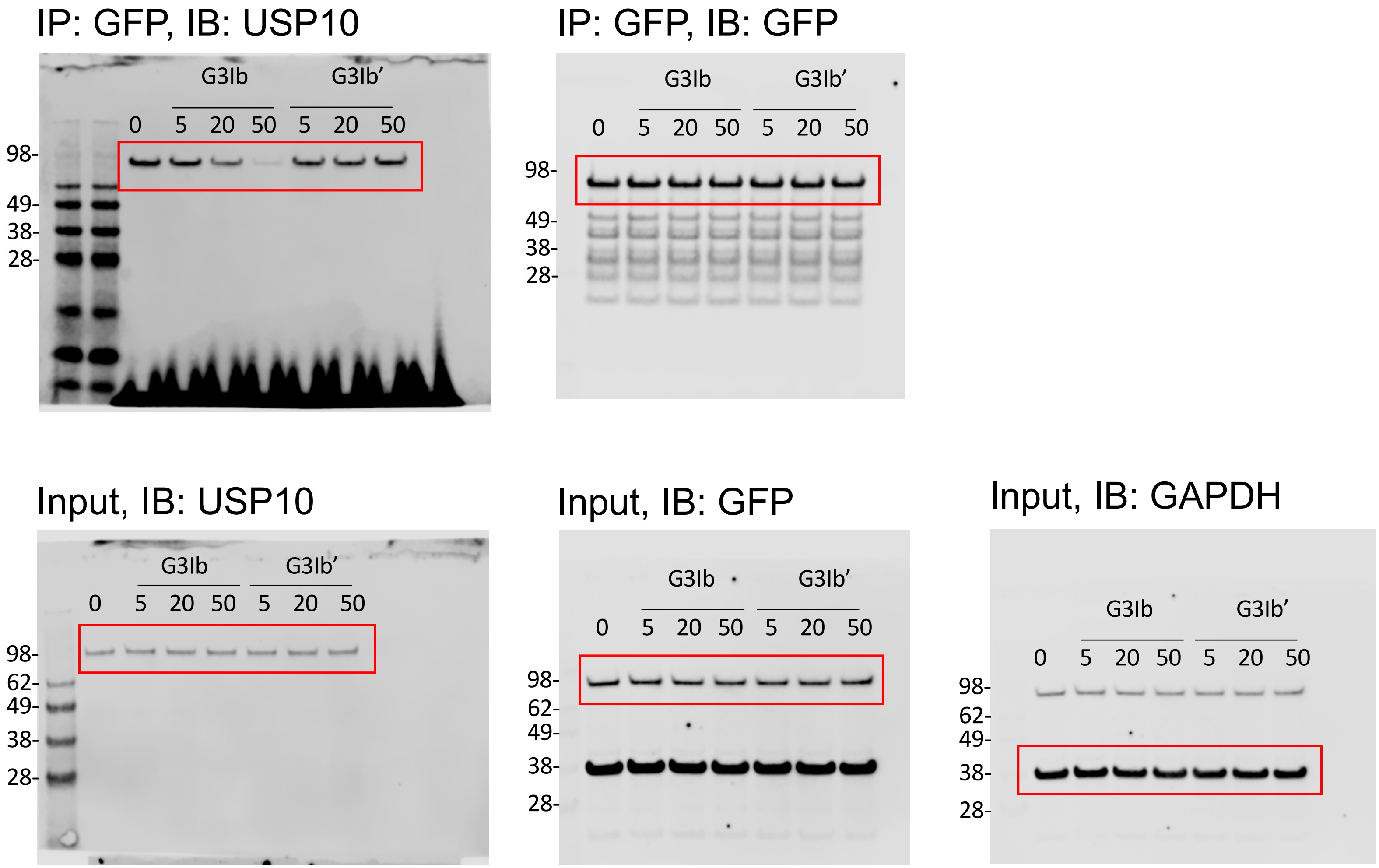
IP: GFP, IB: USP10
G3Ib
G3Ib'
0 5 20 50 5 20 50
98-
49-
38-
28-
IP: GFP, IB: GFP
G3Ib
G3Ib'
0 5 20 50 5 20 50
98-
49-
38-
28-
Input, IB: USP10
G3Ib
G3Ib'
0 5 20 50 5 20 50
98-
62-
49-
38-
28-
Input, IB: GFP
G3Ib
G3Ib'
0 5 20 50 5 20 50
98-
62-
49-
38-
28-
Input, IB: GAPDH
G3Ib
G3Ib'
0 5 20 50 5 20 50
98-
62-
49-
38-
28-

Figure 2E

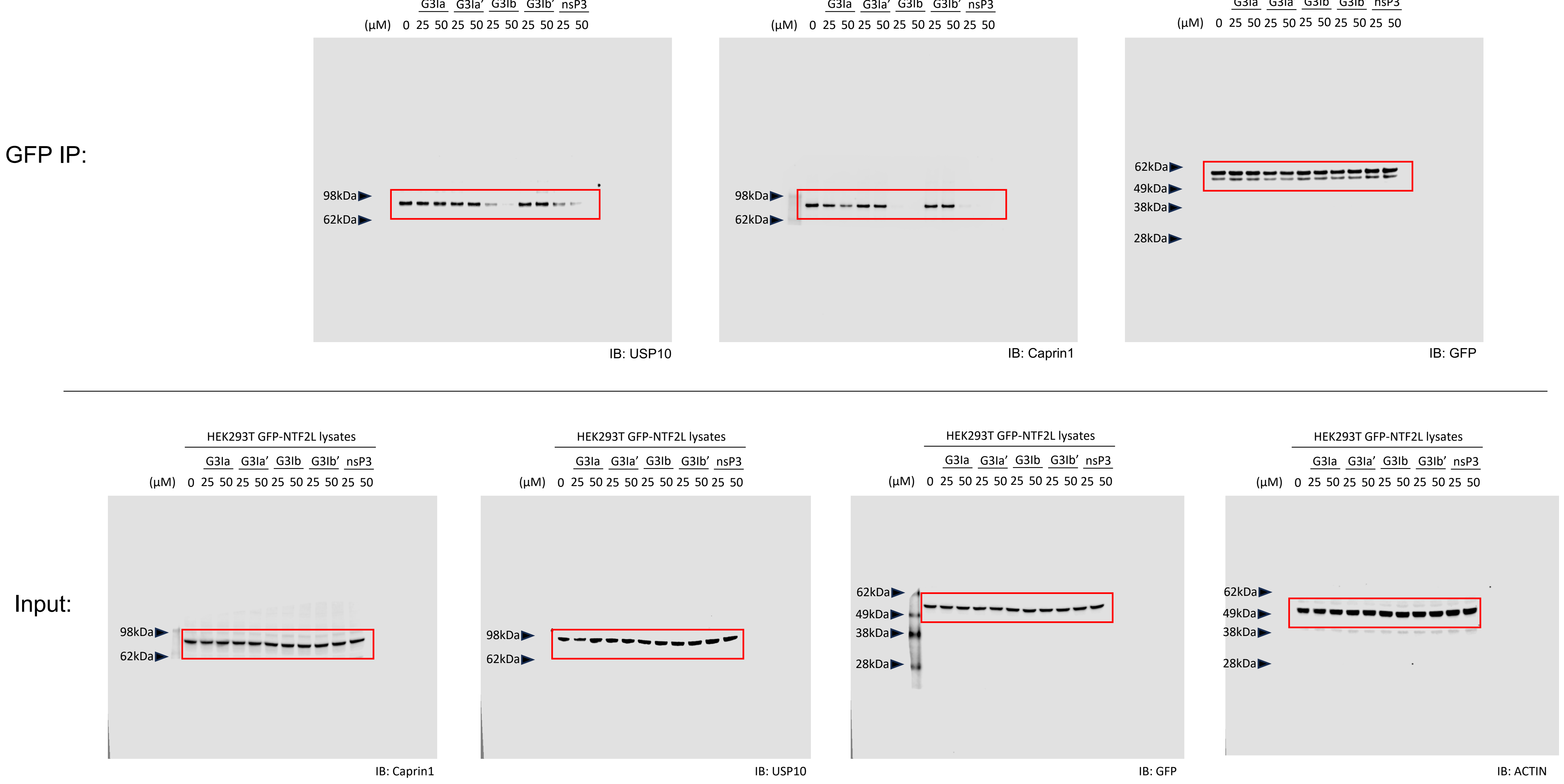
GFP IP:
HEK293T GFP-NTF2L lysates
G3Ia G3Ia’ G3Ib G3Ib’ nsP3
(μM) 0 25 50 25 50 25 50 25 50 25 50
98kDa
62kDa
IB: USP10
HEK293T GFP-NTF2L lysates
G3Ia G3Ia’ G3Ib G3Ib’ nsP3
(μM) 0 25 50 25 50 25 50 25 50 25 50
98kDa
62kDa
IB: Caprin1
HEK293T GFP-NTF2L lysates
G3Ia G3Ia’ G3Ib G3Ib’ nsP3
(μM) 0 25 50 25 50 25 50 25 50 25 50
62kDa
49kDa
38kDa
28kDa
IB: GFP
Input:
HEK293T GFP-NTF2L lysates
G3Ia G3Ia’ G3Ib G3Ib’ nsP3
(μM) 0 25 50 25 50 25 50 25 50 25 50
98kDa
62kDa
IB: Caprin1
HEK293T GFP-NTF2L lysates
G3Ia G3Ia’ G3Ib G3Ib’ nsP3
(μM) 0 25 50 25 50 25 50 25 50 25 50
98kDa
62kDa
IB: USP10
HEK293T GFP-NTF2L lysates
G3Ia G3Ia’ G3Ib G3Ib’ nsP3
(μM) 0 25 50 25 50 25 50 25 50 25 50
62kDa
49kDa
38kDa
28kDa
IB: GFP
HEK293T GFP-NTF2L lysates
G3Ia G3Ia’ G3Ib G3Ib’ nsP3
(μM) 0 25 50 25 50 25 50 25 50 25 50
62kDa
49kDa
38kDa
28kDa
IB: ACTIN
