# Supplementary figures and images for: Identification of small molecule inhibitors of G3BP-driven stress granule formation
Source: J Cell Biol. 2024 Jan 29;223(3):e202308083. doi: 10.1083/jcb.202308083 (PMC10824102; doi:10.1083/jcb.202308083)

Figure S4B

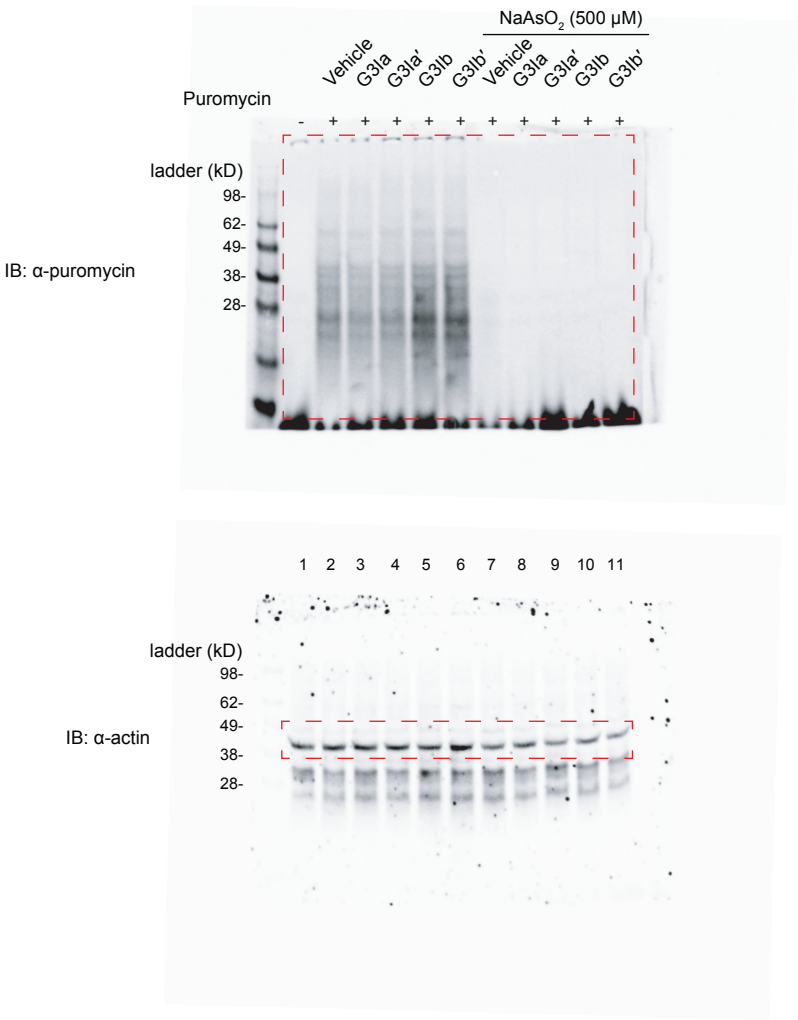

Supplement: SourceData FS4 — is the source file for Fig. S4. [file JCB_202308083_SourceDataFS4.pdf]
